# Language but not TBI History is Associated with Recall Among Hispanic/Latino Adults in the Health and Retirement Study

**DOI:** 10.1002/alz70860_107632

**Published:** 2025-12-23

**Authors:** Alyssa Lauren Lawrence, Carlos E.E. Araujo‐Menendez, Rachel Membreno, Ariana M Stickel

**Affiliations:** ^1^ San Diego State University, San Diego, CA, USA; ^2^ SDSU/UCSD Joint Doctoral Program Clinical Psychology, San Diego, CA, USA

## Abstract

**Background:**

The relationship between traumatic brain injury (TBI) history and risk for Alzheimer's disease (AD) is ambiguous. The Hispanic population faces notable disparities in TBI care and recovery, but research on the potential effects of TBI on memory/cognitive aging is limited. Additionally, existing literature often fails to take into account differences in language of testing which may affect cognitive performance. We hypothesized that individuals without a history of TBI would perform better on a measure of learning and memory than those with a history of TBI, and this would be exacerbated among individuals who were tested in Spanish.

**Methods:**

Participants included Hispanic adults ranging from 34 to 95 years of age (*N* = 207; mean age = 61.7 yrs) from the Health and Retirement Study (HRS) 2014 Core that completed the TBI Module. TBI history was self‐reported (TBI: yes=85). Language was determined by language of testing (English = 108, Spanish=99). Learning and memory were measured using a word list consisting of 10 words, with an immediate and delayed recall. Analyses were conducted using general linear models. Covariates of age, sex, education, language, and TBI history were added to the model, and we tested for an interaction between language and TBI history on cognitive outcomes.

**Results:**

Testing in Spanish was associated with higher scores on immediate and delayed recall on the word list (F(1, 185) = 10.20, *p* <0.01; F(1, 185) = 6.44, *p* <0.01, respectively). There was no association between TBI history and cognitive outcomes, nor were there any significant interactions between TBI history and language on cognition (all *p*'s>0.05).

**Conclusions:**

Contrary to our hypothesis, TBI history groups performed similarly on immediate and delayed recall of the word list measure, and this was not modified by language of testing. Several factors could have contributed to this, such as the fact that TBI history was self‐reported. Given that our data were cross‐sectional, future research should investigate 1) whether the Spanish advantage remains over time and 2) if TBI history may better relate to learning and memory trajectories over time and risk AD.

